# Real-world analysis and future trends of parkinson's disease burden and all-cause mortality in Shanghai Pudong: a population-based study of 3.17 million people

**DOI:** 10.1186/s12889-025-25146-1

**Published:** 2025-11-14

**Authors:** Zheng Luo, Jie Xue, Yan Zhang, Yichen Chen, Aiqing Fan, Ru Liu, Wenchang Jia, Xiaoling Wu, Qizhe Wang, Huihui Lv, Yong Bi, Xiaopan Li

**Affiliations:** 1 Department of Neurology, Shanghai University of Medicine & Health Sciences Affiliated Zhoupu Hospital, 1500 Zhouyuan Rd.,Pudong, Shanghai, 201318 China; 2 Department of Neurology, Zhongshan Hospital, Fudan University, 180 Fenglin Road, Shanghai, China; 3 Office of Scientific Research and Information Management, Centre for Disease Control and Prevention, Pudong New Area, Shanghai, 200136 China; 4 Fudan University Pudong Institute of Preventive Medicine, Pudong New Area, Shanghai, 200136 China; 5 School of Public Health, Fudan University, Shanghai, 200032 China; 6 Sunqiao Community Health Service Center, Pudong New Area, Shanghai, 201210 China; 7 Department of Neurology, Yueyang Hospital of Integrated Traditional Chinese and Western Medicine, Shanghai University of Traditional Chinese Medicine, 110 Ganhe Rd., Shanghai, 200437 China; 8 Department of Health Management Centre, Zhongshan Hospital, Shanghai Medical College of Fudan University, 180 Fenglin Rd. Xuhui, Shanghai, 200032 China

**Keywords:** Parkinson’s disease, Mortality, Years of life lost, Aging, China, Shanghai, Forecasting

## Abstract

**Background:**

To assess all-cause Parkinson's disease (PD)-related and PD-specific mortality trends and years of life lost (YLL) in Shanghai Pudong (3.17 million population) from 2005–2021, and project future burdens through 2035.

**Methods:**

Using population-level mortality data including 4,218 PD-related deaths among 362,558 total deaths, we calculated crude mortality rates (CMR), age-standardized mortality rates (ASMRW), and YLL. Temporal trends were analyzed by the average annual percent change (AAPC) via Joinpoint regression, demographic impacts via decomposition methods, and future projections via ARIMA models.

**Results:**

PD-related CMR and ASMRW were 8.74/100,000 and 2.76/100,000, respectively, with 31,904.41 YLL. PD-specific mortality rates (CMR: 3.76/100,000; ASMRW: 1.25/100,000) accounted for 14,532.50 YLL. Individuals aged ≥ 80 years exhibited highest mortality burden (CMR: 112.29/100,000). Cerebrovascular disease (18.94%) and coronary heart disease (13.54%) were leading comorbidities. Temporal analyses revealed significant annual increases in ASMRW (AAPC = 8.38%, *P* < 0.001) and YLL rates (AAPC = 7.03%, *P* < 0.001), driven predominantly by population aging (AAPC = 30.73%, *P* < 0.001). Projections indicate continued rises in ASMRW (AAPC = 1.49%) and YLL rates (AAPC = 3.49%) through 2035 (*P* < 0.001).

**Conclusions:**

PD imposes increasing mortality burden in Shanghai Pudong, with population aging as the primary driver. Multi-departmental interventions targeting early diagnosis, comorbidity management, and medical infrastructure are urgently needed.

**Trial registration:**

Clinical trial number: not applicable.

**Supplementary Information:**

The online version contains supplementary material available at 10.1186/s12889-025-25146-1.

## Introduction

Parkinson's disease (PD) is a chronic and progressive neurodegenerative disorder characterized by the degeneration of brain neurons over time. It has become a notable contributor to the global neurological disease burden [1, 2]. Within China, PD is recognized as a critical public health issue, with a concerning escalation in disease prevalence. The Global Burden of Disease (GBD) Study 2021 reported an 89.7% increase in age-standardized incidence and a 167.8% increase in prevalence of PD in China [3]. This upward trend is largely attributed to the aging demographic, lifestyle modifications, and environmental influences prevalent in urbanizing cities undergoing rapid demographic transitions [4].

The disease burden of PD in China extends beyond morbidity and mortality rates to encompass Years of Life Lost (YLL), a metric focused on quantifying premature mortality by estimating the gap between actual age at death and the population’s average life expectancy [3]. Notably, China’s age-standardized YLL rate for PD remains notably elevated compared to countries with similar social and demographic structures, reflecting a pronounced challenge in premature mortality associated with the disease [5]. Within China's rapidly urbanizing cities, this burden is amplified by environmental stressors, healthcare accessibility disparities, and lifestyle changes accompanying urban development [6]. Additionally, increasing exposure to environmental neurotoxins, including pesticides (paraquat, rotenone) and industrial solvents (trichloroethylene), may contribute to rising PD mortality rates [7–9]. The distribution of YLL exhibits marked heterogeneity across geographic regions and population subgroups, revealing distinct patterns of health inequity. However, GBD research or national level mortality estimates mainly rely on PD-specific deaths (underlying cause of death), which may underestimate their true burden by ignoring the disease burden of populations with PD who die from other diseases(all-cause of death of patients with PD,or PD-related death) [3, 10, 11]. Understanding these variations in both all-cause and PD-specific mortality outcomes is crucial for developing targeted interventions that address the specific epidemiological profiles of affected communities [12–15].

This study aims to systematically analyze PD's disease burden in a rapidly developing Chinese city, with particular emphasis on mortality patterns, YLL, and modifiable risk factors. Leveraging data from the real world within a 3.17 million population-based framework, the research will elucidate PD's epidemiological trajectory and demographic-specific mortality impacts. The findings are expected to strengthen the evidence base for formulating precision public health strategies to reduce premature mortality and improve survival outcomes for PD patients in China's urbanizing contexts.

## Methods

### Data source

The study compiled data on 4,218 mortality cases linked to PD between 2005 and 2021, utilizing a comprehensive system in Shanghai Pudong, China, that logs demographic and mortality data for a population of 3.17 million individuals (see Additional file 1: Figure S1- S2). The local population data custodians in Pudong furnished us with an exhaustive dataset. We systematically verified and integrated the data to ensure its accuracy and completeness [16]. The system encompasses a broad spectrum of demographic attributes, including age, gender, and the underlying causes of death. Ongoing assessments and data sanitization procedures are conducted to preserve the accuracy and reliability of the registration system.

PD was diagnosed in accordance with the International Classification of Diseases, 10th Revision (ICD-10), specifically codes G20 to G22. PD-related deaths were classified under all-cause mortality when PD was listed as a contributing factor to death. PD-specific mortality was ascertained by clinical practitioners who, after considering the patients' medical histories and pertinent diagnostic tests, confirmed PD as the underlying cause of death [17].

The study protocol was approved by the Ethics Committee of the Health Department in Shanghai Pudong, China. Our research was conducted without any interference with the participants and with stringent adherence to data confidentiality protocols.

### Statistical analyses

Crude mortality rates (CMR) and age-standardized mortality rates (ASMRW) were calculated using Segi's world standard population as reference population, expressed per 100,000 person-years. The Poisson regression model was employed to compare CMR across genders, whereas the Mantel–Haenszel test was utilized for comparing ASMRW. To identify and prioritize causes of premature mortality, YLL and average Years of Life Lost (AYLL) were computed, following the methodology outlined in the Global Burden of Disease Study. The YLL was calculated using the World Health Organization's formula: YLL = KCe^ra^/(r + β)^2^ {e^−(r+β)(L+a)^ [-(r + β)(L + a)−1]-e^−(r+β)a^[-(r + β)a-1]} + [(1-k)/r]*(1-e^−rL^). where *C* represents the age weighting fit with a constant (0.1658), *r* is the discount rate (3%), *a* is the age at death, *β* is the age weighting parameter (0.04), *L* is the average life expectancy at the time of death as determined by the standard reference life table for the Global Burden of Disease (GBD) study14, and *e* is Napier's constant.

Age groups were categorized as follows: 0–4, 5–14, 15–29, 30–44, 45–59, 60–69, 70–79, and 80 years or older. Given the low frequency of PD-related deaths among individuals under 45, trends in age-specific CMR, ASMRW, AYLL, and YLL rates were analyzed for the age groups < 45, 45–59, 60–69, 70–79, and 80 years or older. Temporal trends in CMR, ASMRW, AYLL, and YLL rates were assessed using the Joinpoint Regression Program 4.3.1.0, with results presented as the average annual percent change (AAPC) alongside the corresponding 95% confidence interval (95% CI). The Z-test was applied to ascertain whether the AAPC significantly deviated from zero. Statistically significant AAPCs (*P* < 0.05) were classified as "increases" or "decreases," with "stable" trends indicated for non-significant changes. All statistical analyses were conducted using SPSS (version 21.0) and R (version 3.4.3), with a P value of less than 0.05 considered indicative of statistical significance.

## Results

### Overall mortality and YLL in PD

During the 16-year study period from 2005 to 2021 in Shanghai Pudong New Area, Table 1 presented a detailed summary of mortality data for all-cause and cause-specific deaths attributed to PD. All-cause PD-related deaths (Table 1, Panel A) totaled 4,218 cases, with an average age at death of 79.22 ± 8.24 years. Males comprised 57.06% (*n* = 2,407) and females 42.94% (*n* = 1,811) of deaths, with males dying slightly younger (79.22 ± 8.11 vs 80.84 ± 8.32 years). The CMR was 8.74 per 100,000 population and ASMRW was 2.76 per 100,000. The aggregate YLL reached 31,904.41 years (rate: 66.09 per 100,000; average: 7.56 years per individual). The most common comorbidities were cerebrovascular disease (18.94%), coronary heart disease (13.54%), and chronic lower respiratory diseases (6.35%).

**Table 1:** Baseline characteristics of Parkinson's disease-related or specific deaths

Characteristic	Deaths (n, %)	Age at years (Mean ± SD)		Age at years (Median)	CMR (/10^5^)	ASMRW (/10^5^)	YLL (years)	YLL rate (/10^5^)	AYLL (year/person)
Panel A: All cause of death with Parkinson's disease (PD-related death)									
Gender									
Male	2407 (57.06)		79.22 ± 8.11	79.93	9.99	3.68	17,725.62	73.59	7.36
Female	1811 (42.94)		80.84 ± 8.32	81.92	7.49	2.05	14,178.79	58.62	7.83
Total	4218 (100.00)		79.22 ± 8.24	80.83	8.74	2.76	31,904.41	66.09	7.56
The top 3 disease types in all causes of death, excepts Parkinson's disease									
Cerebrovascular disease (I60-69)	799 (18.94)		80.97 ± 7.27	81.74	1.66	0.50	5647.73	11.70	7.07
Coronary Heart diseases (I20-25)	571 (13.54)		83.05 ± 7.16	83.86	1.18	0.34	3679.37	7.62	6.44
Chronic lower respiratory diseases (J40-47)	268 (6.35)		80.59 ± 7.62	81.54	0.56	0.17	1919.57	3.98	7.16
Panel B: Parkinson's disease specific death									
Gender									
Male	1024 (56.51)		78.03 ± 8.70	79.06	4.26	1.61	8032.56	33.35	7.84
Female	788 (43.49)		79.90 ± 8.68	81.03	3.26	0.93	6499.94	26.87	8.25
Total	1812 (100.00)		78.84 ± 8.74	79.92	3.76	1.24	14,532.50	30.10	8.02
The top 3 co-morbidity in underlying causes of death									
Other diseases of the respiratory system (J95-99)	675 (37.25)		79.31 ± 8.45	80.41	1.40	0.46	5233.09	10.84	7.75
Hypertensive diseases (I10-15)	419 (23.12)		81.14 ± 7.57	81.98	0.87	0.26	2955.50	6.12	7.05
Influenza and pneumonia (J10-18)	247 (13.63)		78.82 ± 8.97	81.98	0.51	0.17	1957.25	4.05	7.92

*ASMRW* age-standardized mortality rate by Segi’s world standard population, *CMR* crude mortality rate, *YLL* years of life lost

PD-specific deaths (Table 1, Panel B) accounted for 1,812 cases, with an average age at death of 78.84 ± 8.74 years. Males represented 56.51% (*n* = 1,024) and females 43.49% (*n* = 788), with males dying younger (78.03 ± 8.70 vs 79.90 ± 8.68 years). The CMR was 3.76 and ASMRW was 1.246 per 100,000 population. The YLL burden totaled 14,532.50 years (rate: 30.10 per 100,000; average: 8.02 years per individual). The leading comorbidities were other respiratory system diseases (37.25%), hypertensive diseases (23.12%), and influenza and pneumonia (13.63%).

### Gender- and age-specific differences in the burden of all-cause and cause-specific PD

**Fig. 1:**
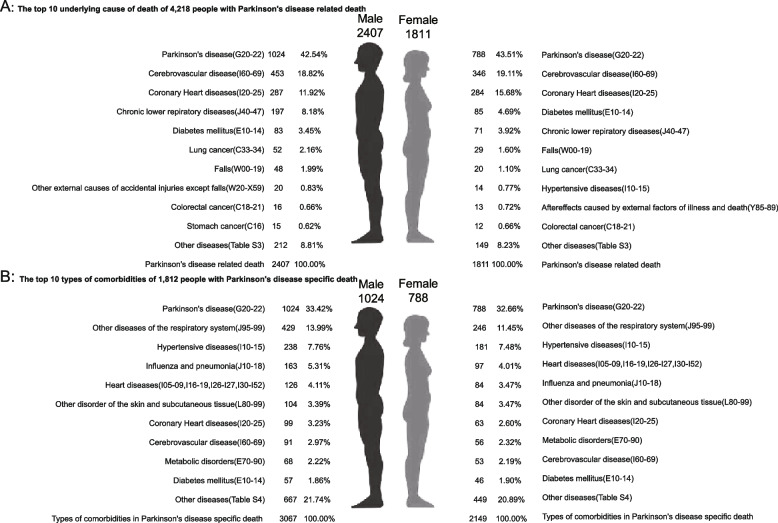
Top ten all causes of death of people who died of/with Parkinson's disease in Shanghai Pudong, China, 2005–2021

The analysis revealed distinct mortality patterns across gender and age groups. Figure 1 demonstrates the top 10 contributing causes of death among individuals with PD-related mortality and the comorbidities associated with PD-specific deaths (see Additional file 1: Table S1-S2).

Gender-specific mortality patterns revealed notable differences between males and females. Among 4,218 PD-related deaths (Fig. 1, Panel A), males primarily died from cerebrovascular disease (18.82%), coronary artery disease (11.92%), and chronic obstructive pulmonary disease (8.18%). In females, cerebrovascular disease (19.11%) and coronary artery disease (15.68%) were most common, followed by diabetes mellitus (4.69%).

For PD-specific deaths (Fig. 1, Panel B), other respiratory diseases predominated in both genders (males: 13.99%; females: 11.45%), followed by hypertension (males: 7.76%; females: 7.48%) and influenza/pneumonia in males (5.31%) versus heart disease in females (4.01%).

Age-related mortality burden was heavily concentrated in older age groups. Table 2 presents mortality and YLL across age cohorts, revealing a clear age-dependent pattern. For all-cause PD-related deaths (Table 2, Panel A), a particularly striking pattern emerged when transitioning from the 60–69 age group (378 cases, 8.96%) to the 70–79 age group (1,469 cases, 34.83%), with deaths nearly quadrupling that suggests the 8th decade of life as a critical threshold for PD mortality risk. The 80 + age group recorded 2,288 cases (54.24% of total mortality) with the highest CMR (112.29 per 100,000) and greatest YLL (11,728.61 years; rate: 575.60 per 100,000), averaging 5.13 years per person. Conversely, the youngest group (< 45 years) had minimal mortality (seven cases; CMR: 0.03 per 100,000) but substantial individual loss (averaging 23.91 YLL years per person).

**Table 2:** Number of Parkinson's disease-related or specific deaths, CMR, YLL, and YLL rate by age group

Age group (years)	Deaths (N)	Proportion (%)	CMR (/10^5^)	YLL (years)	YLL rate (/10^5^)	AYLL (years/person)
Panel A: Related deaths						
< 45 years	7	0.17	0.03	167.40	1.28	23.91
45–59 years	76	1.80	0.62	1398.50	11.33	18.40
60–69 years	378	8.96	5.57	5271.33	77.69	13.95
70–79 years	1469	34.83	40.99	13338.56	372.23	9.08
≥ 80 years	2288	54.24	112.29	11728.61	575.60	5.13
Total	4218	100.00	8.74	31904.41	66.09	7.56
Panel B: Specific deaths						
< 45 years	6	0.33	0.05	140.42	1.28	23.40
45–59 years	50	2.76	0.41	915.36	7.42	18.31
60–69 years	197	10.87	2.90	2761.18	40.69	14.02
70–79 years	661	36.48	18.45	6065.31	169.26	9.18
≥ 80 years	898	49.56	44.07	4650.22	228.22	5.18
Total	1812	100.00	3.76	14532.50	30.10	8.02

*CMR* crude mortality rate, *YLL* years of life lost

For PD-specific deaths (Table 2, Panel B), a similar dramatic escalation was observed from the 60–69 age group (197 cases, 10.87%) to the 70–79 age group (661 cases, 36.48%), with cases more than tripling, reinforcing the critical nature of this age transition in PD mortality. The oldest group similarly dominated with 898 cases (49.56% of total deaths) and CMR of 44.07 per 100,000. They accumulated 4,650.22 YLL years (rate: 228.22 per 100,000), averaging 5.18 years per person. The youngest group had six deaths, each averaging 23.40 YLL years.

### Temporal trends in PD mortality and YLL

PD-attributed mortality showed a consistent upward trajectory, increasing from 0.56% in 2005 to 1.70% in 2021. PD-specific deaths rose substantially, comprising an average of 42.96% of all PD-related fatalities (see Additional file 1: Table S3 and Figure S3).

**Fig. 2:**
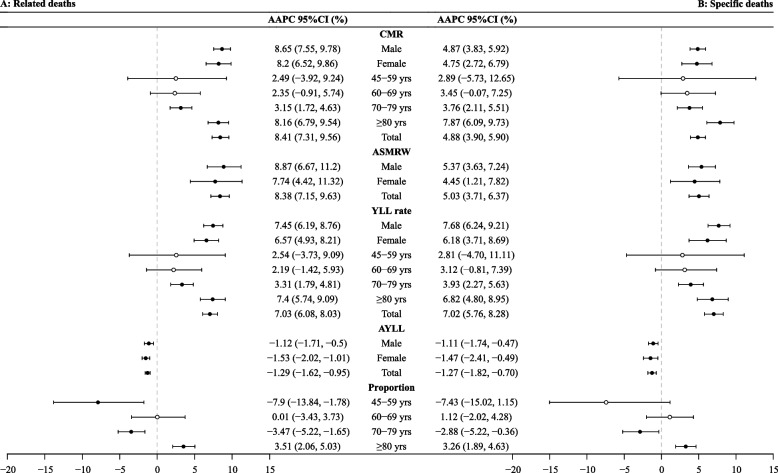
The trends in CMR, ASMRW, and YLL of persons with death from Parkinson's disease in Shanghai Pudong, China, 2005–2021. Notes: CMR, crude mortality rate (per 100,000); ASMRW, age-standardized mortality rate by Segi’s world standard population (per 100,000); YLL, year of lost. AAPC, average annual percent change; CI, confidence interval

The evolution of PD-related mortality and YLL rates across age groups and genders is captured in Fig. 2, which reveals significant temporal changes (see Additional file 1: Table S4-S7). For all-cause PD-related deaths (Fig. 2, Panel A), CMR, ASMRW, and YLL rates increased significantly among males, females, and overall population (all *P* < 0.05), while average YLL decreased from 2005 to 2021 (*P* < 0.05). CMR and YLL rates showed significant increases among those aged over 70 (all *P* < 0.05).

The demographic shift toward older age groups was particularly striking, with the proportion of deaths among individuals over 85 years increasing significantly (*P* < 0.05), while deaths among those aged 45–59 and 70–79 declined (all *P* < 0.05). For PD-specific deaths (Fig. 2, Panel B), similar trends emerged, with increases particularly pronounced in older age groups and males. However, deaths among those aged 45–59 remained stable (*P* > 0.05).

PD-related and cause-specific AYLL between genders showed fluctuations rather than consistent trends, suggesting dynamic changes in disease impact over time (see Additional file 1: Table S8).

### Quantitative impact of population aging on CMR by decomposition methods

**Fig. 3:**
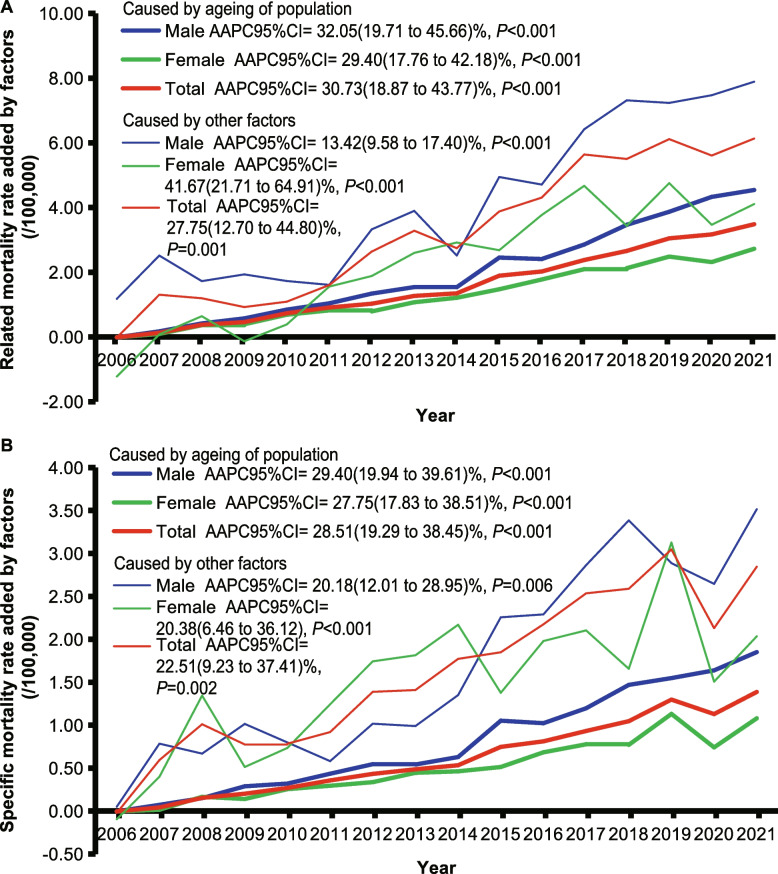
The increased rates of death caused by aging and other factors during the period from 2006 to 2021 compared with the crude mortality rate of Parkinson's disease during 2005 in Shanghai Pudong, China. Notes: AAPC, average annual percent change; CI, confidence interval

The contribution of population aging to rising PD mortality rates is clearly demonstrated in Fig. 3, which shows trends and impacts from 2006 to 2021, categorized by gender and contributing factors.

For all-cause PD-related mortality rates (Fig. 3, Panel A), both males and females experienced marked upward trends due to population aging and other factors. Population aging emerged as a significant driver of increased PD-related mortality rates, with the annual increase in CMR attributable to population aging showing AAPCs of 30.73% overall, 32.05% for males, and 29.40% for females (all *P* < 0.001). This indicates that the contribution of aging to PD-related mortality grew by approximately 30% per year. Compared to 2005 CMR baseline, the proportion of CMR growth attributed to population aging from 2006 to 2021 varied from 9.03% in 2007 to 40.20% in 2010.

For PD-specific mortality rates (Fig. 3, Panel B), population aging remained a substantial driver with the aging-attributable CMR increase demonstrating AAPCs of 28.51% overall, 29.40% for males, and 27.75% for females (all *P* < 0.001). This means that aging's contribution to PD-specific mortality increased by approximately 28% annually. Relative to 2005 baseline CMR, the added value due to population aging ranged from 6.65% in 2007 to 34.65% in 2020 (see Additional file 1: Table S9).

### Longitudinal prediction analysis of PD mortality and YLL rates

**Fig. 4:**
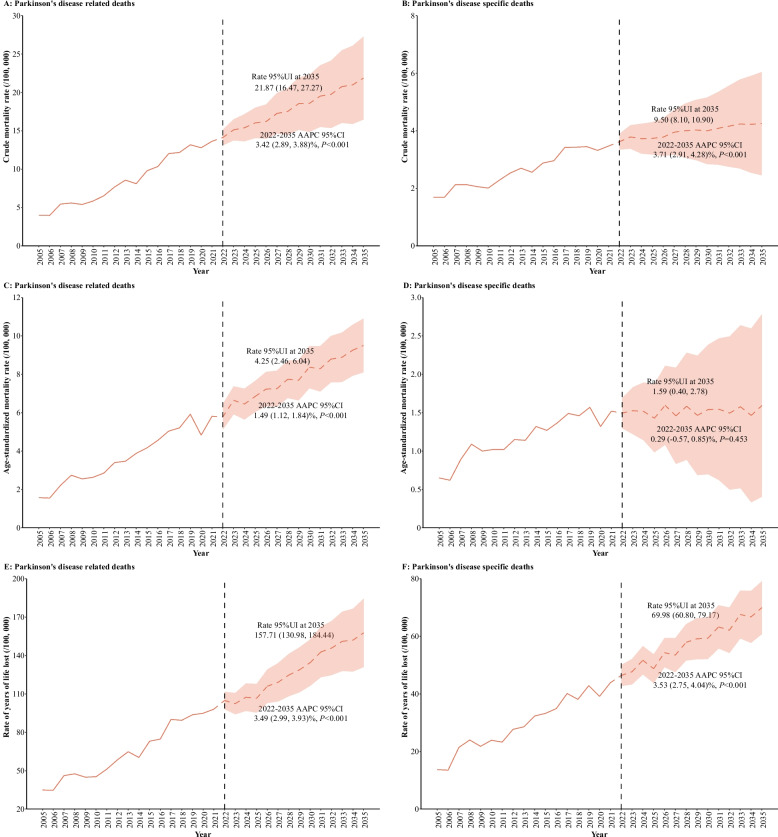
The crude mortality rate, age-standardized mortality rate, and rate of year of life lost from Parkinson's disease related/specific deaths in Shanghai Pudong assessed from 2005 to 2021, alongside forecasted values for the period between 2022 and 2035. Notes: CMR, crude mortality rate (per 100,000); ASMRW, age-standardized mortality rate by Segi’s world standard population (per 100,000); YLL, year of lost. AAPC, average annual percent change; CI, confidence interval

Future projections reveal an alarming trajectory for PD mortality burden. Figure 4 outlines temporal trends in CMR, ASMRW, and YLL rates from 2005 to 2021, with projections extending to 2035 (see Additional file 1: Table S10).

For all-cause PD-related deaths, CMR is expected to rise dramatically from 4.01 to 21.87 per 100,000 by 2035, while PD-specific deaths are projected to increase from 1.57 to 9.50 per 100,000 over the same period (all *P* < 0.05) (Fig. 4, Panel A and B). ASMRW for PD-related deaths (Panel C) is anticipated to climb from 1.69 to 4.25 per 100,000 (*P* < 0.05), while PD-specific deaths are projected to increase from 0.65 to 1.59 per 100,000 by 2035 (*P* = 0.453) (Fig. 4, Panel C and D). Additionally, the YLL rates were also anticipated to rise significantly, with PD-related deaths projected to increase from 34.87 to 157.71 per 100,000, and PD-specific deaths from 13.70 to 69.98 per 100,000 by 2035 (all *P* < 0.05) (Fig. 4, Panel E and F).

## Discussion

This study uncovers notable variances in the all-cause and cause-specific burdens of PD within the developed urban setting of Shanghai Pudong, China. Our data indicate higher CMR and ASMRW for PD-related deaths compared to PD-specific deaths, which underscores the considerable disease burden within this urbanizing locale. When juxtaposed with the Global Burden of Disease Study 2021 estimates for China, our findings suggest an elevated disease burden at the local level [3]. Such discrepancies could be attributed to a multitude of factors, including environmental risk factors such as heightened exposure to industrial pollutants and pesticides, which are particularly prevalent in the rapidly industrializing region of Shanghai Pudong [15]. Furthermore, disparities in healthcare access, including variances in the availability and quality of neurologist services, early diagnosis initiatives, and treatment adherence, may also contribute to the heightened burden [18]. The co-occurrence of comorbidities, notably cerebrovascular disease, coronary heart disease, and chronic lower respiratory diseases among PD-related deaths, further complicates PD management and exacerbates the overall burden [19–21]. These elements, in conjunction with an aging demographic and the rising prevalence of PD with increasing age, culminate in a more pronounced disease burden in Shanghai Pudong than that depicted by national or global estimates. This highlights the imperative for targeted interventions and a more sophisticated comprehension of local disease dynamics [22–24].

To contextualize our findings, we compared Parkinson's disease burden with other neurodegenerative diseases using global and national data. Globally, the GBD 2021 study showed Parkinson's disease had greater age-standardized disability-adjusted life-year (DALY) rate growth (10.0%) than Alzheimer's disease and other dementias (1.7%) from 1990–2021 [25]. In China, Zhang et al. [26] found Parkinson's disease prevalence growth (678.89%) substantially exceeded Alzheimer's disease and other dementias (322.18%). The study also showed that for DALYs, Alzheimer's disease and other dementias had the largest absolute increase (272.71%), followed by Parkinson's disease (215.25%) and cerebrovascular diseases (136.05% for ischemic stroke, 20.57% for intracerebral hemorrhage). These findings indicate Parkinson's disease represents one of the most rapidly expanding neurodegenerative disease burdens globally and in China, supporting our detailed local analysis.

First, the elevated incidence of PD-related deaths and the corresponding YLL underscore the profound impact of PD on mortality rates and life expectancy. The mean age at death, nearing 80 years, aligns with the global demographic shift towards an aging population [27, 28]. The considerable YLL is indicative of PD's chronic nature and its adverse impact on quality of life, underscoring the necessity for early intervention strategies and enhanced disease management to mitigate PD's burden [29]. The increased CMR and ASMRW suggest an escalating health concern, underscoring the critical need for public health initiatives focused on PD [30, 31].

Second, the gender distribution of PD-related mortality, characterized by a higher prevalence among males, may reflect inherent biological differences in disease susceptibility or health-seeking behaviors [32, 33]. The marginal difference in mean ages at death could be ascribed to a multitude of factors, including genetic predispositions, environmental exposures, and lifestyle choices. A comprehensive understanding of these disparities is essential for the development of targeted interventions aimed at reducing PD-related mortality [34]. Furthermore, the observed gender disparity underscores the necessity for gender-specific research endeavors to elucidate the underlying causes of this distribution and to inform gender-tailored treatment strategies [35, 36].

Third, the high prevalence of significant comorbidities among PD patients underscores the intricate nature of PD management; these conditions can intensify disease symptoms and complicate therapeutic interventions. The prominence of cerebrovascular disease and coronary heart disease as leading comorbidities suggests a common risk factor profile shared with PD, encompassing age, hypertension, and lifestyle elements [21]. This necessitates a holistic management strategy that concurrently addresses PD and its associated comorbidities to enhance patient outcomes [13]. The elevated incidence of chronic lower respiratory diseases further underscores the necessity for respiratory support in PD patients, particularly in the advanced stages of the disease [13]. Focusing on PD-specific deaths offers a more precise depiction of the disease's direct impact, independent of other comorbidities. The mean age at death and the corresponding YLL serves as pivotal indicators for evaluating the efficacy of PD management strategies [37]. The observed lower mean age at death in PD-specific cases compared to all PD-related deaths suggests that PD-specific mortality may manifest at an earlier disease stage, highlighting the imperative for early diagnosis and intervention [38]. The PD-specific YLL also suggests the potential to enhance life expectancy through targeted therapeutic approaches and supportive care [39]. The disparities in YLL rates between PD-related and PD-specific deaths highlight the critical importance of distinguishing between these categories when devising public health strategies [17].

Finally, given the escalating burden of PD-specific mortality and the heightened incidence of PD-related deaths, there is an immediate imperative for intervention strategies. Such strategies should encompass early diagnostic initiatives, enhanced healthcare accessibility, and targeted public health campaigns focused on risk factor mitigation, including the promotion of physical activity and the management of comorbid conditions [12]. Additionally, reducing environmental exposures to neurotoxins such as pesticides and industrial solvents is crucial in tackling the increasing burden of PD, as these represent important modifiable risk factors [7–9]. The anticipated surge in PD-related mortality and YLL rates signals an alarming trend, underscoring the escalating public health challenge that PD presents. This forecast accentuates the critical need for investment in research aimed at refining therapeutic outcomes and instituting preventive measures [40]. The observed upward trajectory in CMR and ASMRW may indicate a deficiency in the capacity of existing healthcare frameworks to effectively manage the expanding PD patient population [41]. The escalating YLL rate suggests a potential diminution in the quality of life for future PD sufferers, thereby accentuating the necessity for advanced palliative care and support systems [29]. These forthcoming projections can serve as a guide for healthcare planning and resource distribution, facilitating better preparedness for the projected augmentation in PD burden.

This study's strengths are evident in its population-based approach, thorough data collection processes, and the incorporation of both all-cause and cause-specific mortality data. Nonetheless, it is not without limitations: these include the potential for misclassification of PD-related deaths and a dependence on hospital discharge records. Such records may fail to account for all PD cases within the community, although efforts have been made to meticulously examine instances of unexplained deaths within local households [42]. Additionally, the generalizability of these findings to regions with distinct healthcare systems and varying PD prevalence rates should be approached with caution [43]. Our study's focus on Parkinson's disease alone, without comparison to other neurodegenerative diseases, limits broader contextual interpretation of disease burden priorities. A further limitation is that our data analysis extends only through 2021. However, the inevitable delay in data availability is understandable, and our 16-year observation period (2005–2021) provides a solid foundation for trend analysis and projection modeling through 2035.

## Conclusions

This study provides an in-depth examination of both all-cause and cause-specific PD burdens within a dynamic urban environment in China, revealing variations in mortality and YLL rates. The results underscore an urgent need for tailored interventions that consider the gender, age, and disease-specific nuances of PD's impact. Anticipating a rise in PD-related burden by 2035, it is imperative to implement preemptive public health strategies aimed at alleviating the effects of this progressive neurodegenerative condition on affected individuals and the broader healthcare infrastructure.

## Supplementary Information

Supplementary Material 1.

## Data Availability

The datasets used and analysed during the current study are available from the corresponding author on reasonable request.

## Abbreviations

AAPC: Average Annual Percent Change
ASMRW: Age-Standardized Mortality Rate by Segi's World Standard Population
AYLL: Average Years of Life Lost
CI: Confidence Interval
CMR: Crude Mortality Rate
DALY: Age-standardized disability-adjusted life-year
GBD: Global Burden of Disease
ICD-10: International Classification of Diseases, 10th Revision
PD: Parkinson's Disease
WHO: World Health Organization
YLL: Years of Life Lost
