# Supplementary figures and images for: Real-world analysis and future trends of parkinson's disease burden and all-cause mortality in Shanghai Pudong: a population-based study of 3.17 million people
Source: BMC Public Health. 2025 Nov 14;25:3960. doi: 10.1186/s12889-025-25146-1 (PMC12619288; doi:10.1186/s12889-025-25146-1)

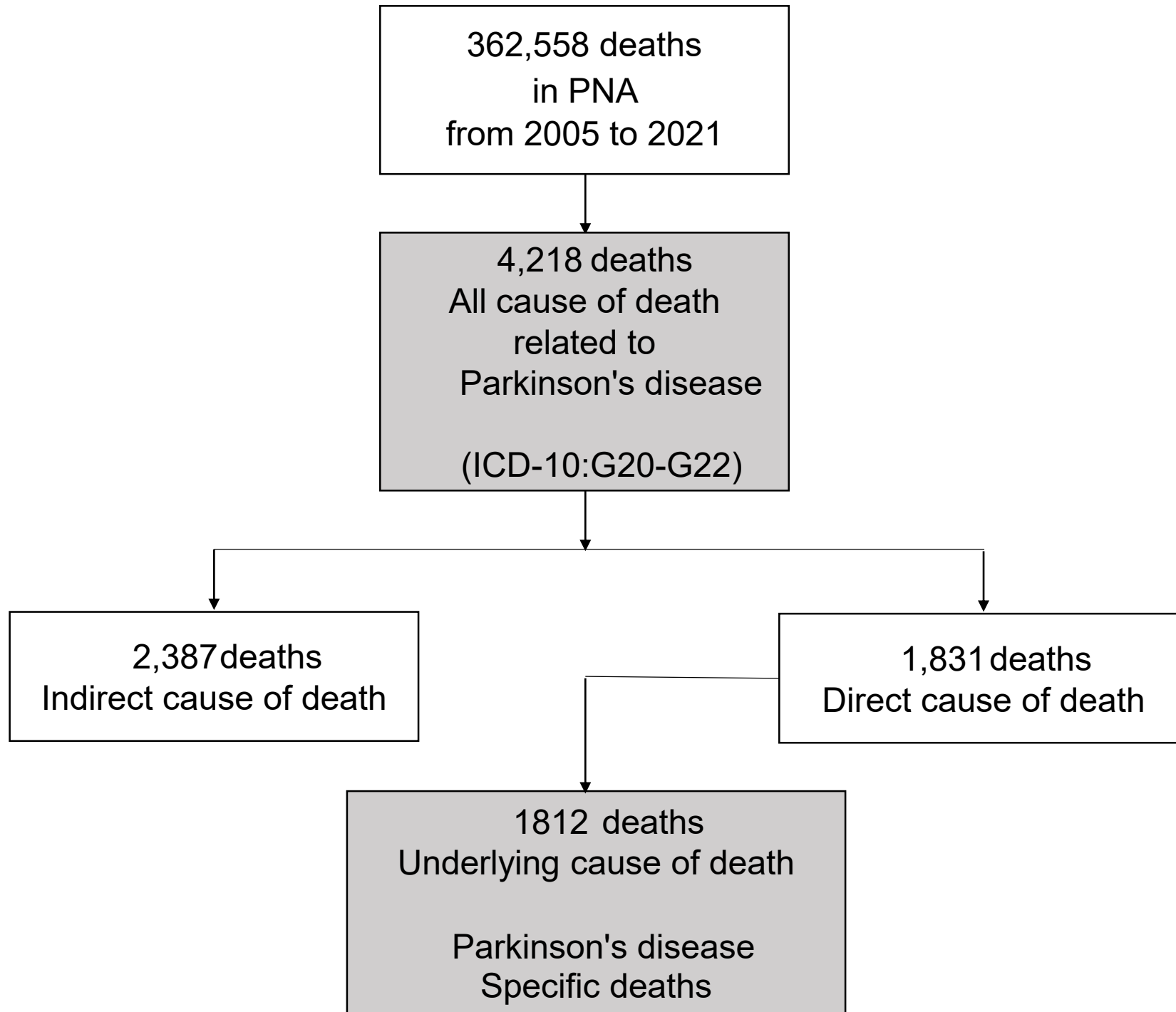

Supplement: Supplementary file 1 — Supplementary Material 1. [file 12889_2025_25146_MOESM1_ESM.zip › Supporting File/Figure S1.pdf]

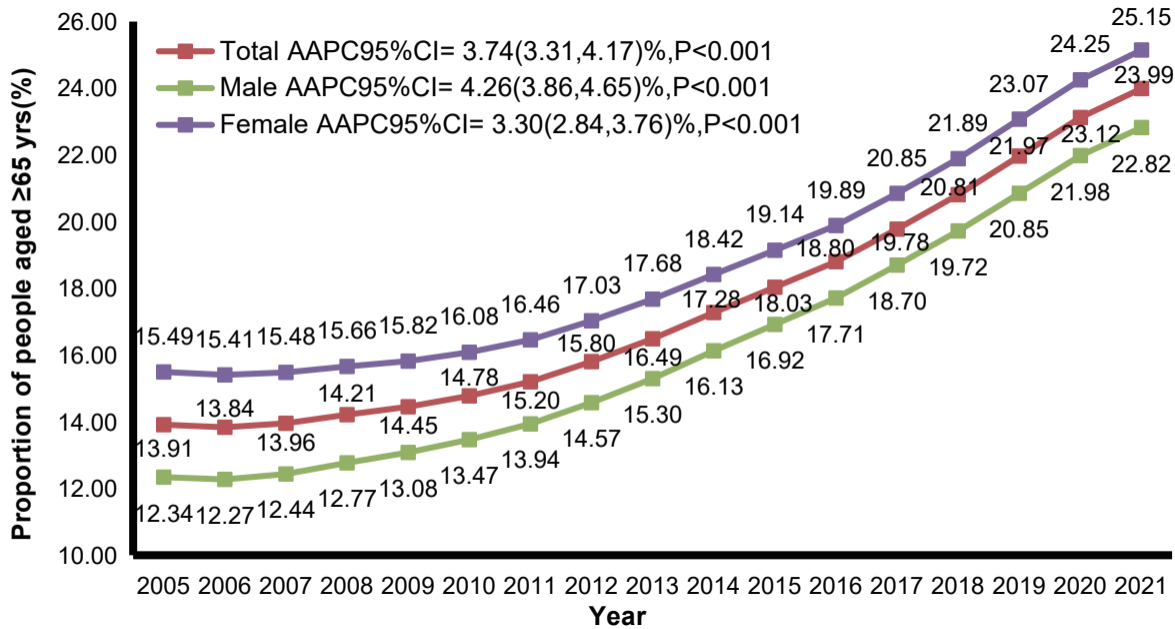

Supplement: Supplementary file 1 — Supplementary Material 1. [file 12889_2025_25146_MOESM1_ESM.zip › Supporting File/Figure S2.pdf]

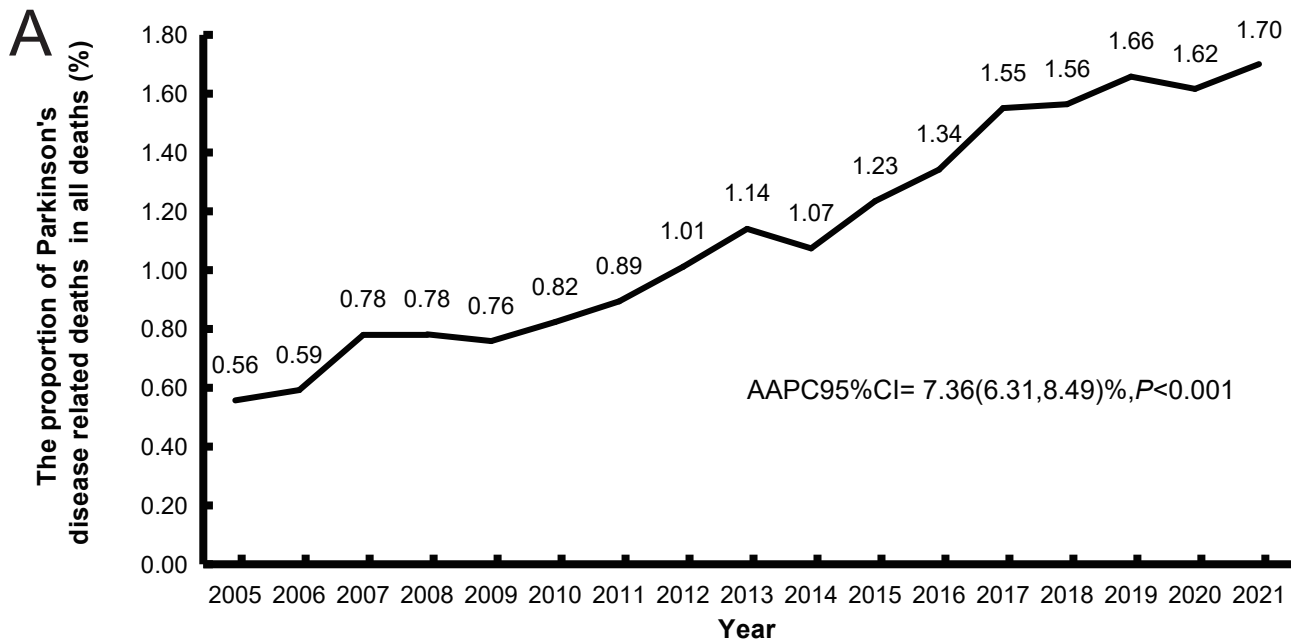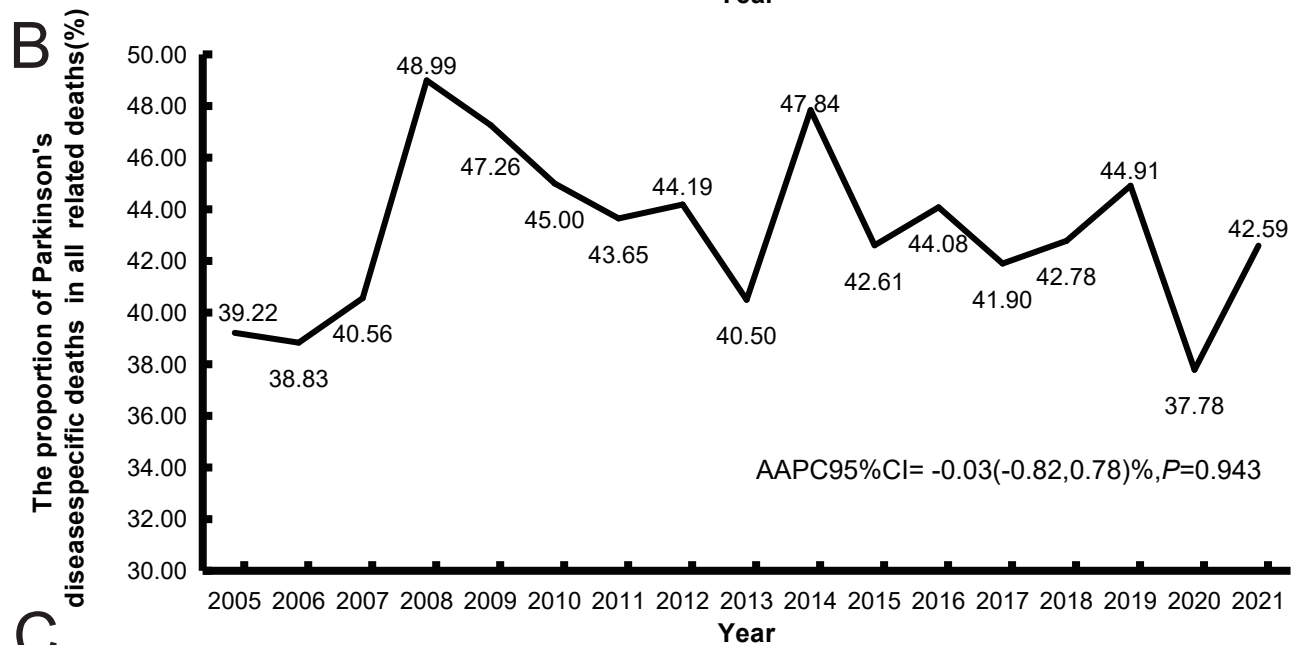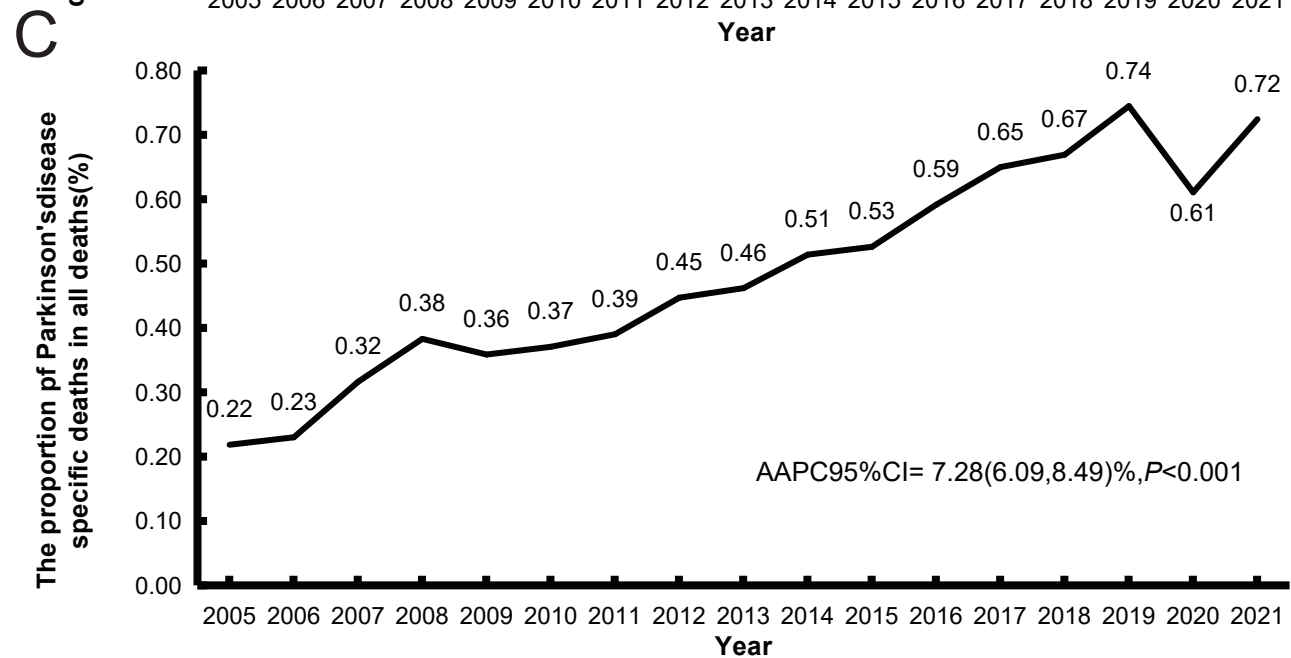

Supplement: Supplementary file 1 — Supplementary Material 1. [file 12889_2025_25146_MOESM1_ESM.zip › Supporting File/Figure S3.pdf]
